# Targeting Tyrosine Kinases in Cancer: Lessons for an Effective Targeted Therapy in the Clinic

**DOI:** 10.3390/cancers11040490

**Published:** 2019-04-06

**Authors:** Adriano Angelucci

**Affiliations:** Department of Biotechnological and Applied Clinical Sciences, University of L’Aquila, L’Aquila 67100, Italy; adriano.angelucci@univaq.it

The 21st century has finally seen the full achievement of targeted therapy to treat cancer with the promise of lower side effects and improved efficacy with respect to cytotoxic chemotherapeutics. Today, in oncological practice, we have numerous antitumoral “bullets” with 114 approved drugs that target 128 different cancer-associated proteins [1,2]. Tyrosine kinases (TKs) represent the most abundant class of targets comprising 52 different proteins and 41 approved drugs (Figure 1). Searching for the terms “tyrosine kinase” and “cancer” in ClinicalTrial.gov database results in about 300 recruiting clinical trials, stating the actuality of this topic [3]. This is not a surprise for oncologists, in fact TKs represent an ideal target for different reasons. First, TKs modulate the intracellular signals that activate survival, proliferation and migration of cells. Indeed, many TKs have a driver role in carcinogenesis and cancer progression. In addition, the catalytic mechanism utilizing ATP renders easy the synthesis of small molecules able to block TK enzymatic activity. Numerous effective TK inhibitors (TKis) have reached the clinic, with some cancer types that have a rich repertoire of TKs; for example, more than ten drugs have been approved for lung cancer, while leukemia represents the cancer with the highest number of targeted TKs (Figure 2). What have we learned about the role of TKs in cancer therapy and what are the unsolved questions about their utilization as curative agents? Let’s examine some hot aspects in the current discussion.

1-Patient selection: Preclinical studies have often shown that TKs play an important role in sustaining basic functions in cancer biology. This evidence encouraged oncologists to design clinical trials that did not consider the actual expression of TKs in the patient. On the contrary, results from clinical trials have clearly demonstrated that the assessment of the expression and activity of the target is fundamental in order to predict the efficacy of the drug. If a therapeutic prediction based on the oncogenic activation of the target is easily achievable for certain hematological tumors with few genetic alterations (e.g., BCR-ABL transcript present in chronic myeloid leukemia), unfortunately, it is not equally practicable in high heterogeneous solid tumors. A striking example of the real use of this approach in solid tumors is the analysis of HER2 overexpression that is propaedeutic for the use of trastuzumab in the treatment of breast carcinoma. In fact, although all members of ERBB family could play a promotion role in cancer, important therapeutic benefits have been described only targeting overexpressed family member or in presence of an oncogenic variant form. It is the case of ERBB4 variant that Donoghue et al. individuated as an independent prognostic marker for glioblastoma subpopulations with poorer prognosis [4]. An open question is whether members of ERBB family other that EGFR, also when non-overexpressed, could exert an underestimated driver role in aberrant activation of EGFR pathway, thus influencing response to EGFR inhibitors.

Although a precision definition of gene expression profile in single patient is important in determining therapeutic cohorts, an effective strategy of patient selection needs more solid evidence about predictive markers. Kozaki et al. utilized a wide panel of hematopoietic cell lines in order to investigate the response to BTK inhibitor, and then they explored the most effective combination therapy in order to avoid BTKi resistance [5]. This type of studies, when associated with the analysis of molecular background, could be fundamental in identifying biomarkers that would predict responses in different cancer subtypes.

2-Selectivity for the target: Single target therapies have shown activity for only few cancer types. The current data support the hypothesis that in the majority of cancers agents targeting multiple signalling pathways are more effective than single targeted therapy. Among 32 TKi small molecules approved for therapy only about 30% could be considered single targeted. The median number for these drugs is 3 targeted TKs. However, it is questionable if multi-TK inhibition should be achieved by a single multitarget inhibitor or by different selective inhibitors. Currently the most accredited answer is the use of combination therapy. In fact, the high number of deregulated pathways and the inter-patient heterogeneity in TK profiles render less feasible the use of a single multi-targeted TKi. Another problem for the utilization of a single multi-kinase inhibitor is that it should preferentially have the same high activity against different targets. Simiczyjew et al. demonstrated that the combination of two, relatively target-restricted TKis, could be effective in very aggressive breast cancer subtype, where HER2 cannot be used as single target [6]. These studies help to understand also if multitarget therapy could prevent resistance due to signalling crosstalk. In fact, the precise definition of signalling pathways associated with resistance to molecularly-targeted therapies can reveal new targets. This is the case of TYRO3, that has revealed non-overlapping function with the other member of the TAM family, AXL, (targeted by gilteritinib, approved for leukemia). Smart et al. describe the signalling pathways downstream of TYRO3, that are potentially associated with TKi resistance and discuss the potential role of agents targeting it with greater selectivity [7]. Effective multitarget combination therapies could benefit also from the use of drugs that inhibit non-TK targets but sensitize to TKis. In this context, Mi et al. discussed how oxidative status in leukemia could cooperate with oncogenic potential of STAT5 [8]. Targeting ROS provides therapeutic benefit in overactivated STAT5 leukemia, offering new choices in determining treatment protocols safer than current ones based upon long-term high dose monotherapy. Innovative combination therapies could derive also by modulating tyrosine phosphatases (PTPs) expression. PTPs represent the dark side of oncogenic activation by tyrosine phosphorylation and their activity is frequently associated with inhibition of cellular processes downstream of TK pathways. Huang et al. evidenced the importance of PTPs in hepatocellular carcinoma progression, suggesting that for those PTPs that act as tumor suppressors it could be hypothesized a combination therapy with TKis using PTP agonists or restoring their expression [9].

3-mutations: Despite the increase in overall survival allowed by TKis in many cancers, drug resistance onset based upon aminoacidic substitution may generate a challenging therapeutic hurdle. Investigation of structural mechanism of action and the evaluation of TKi activity against the most frequent mutant forms of the target is fundamental to overcome this type of resistance. Ponatinib, third-generation BCR-ABL TKi, represents a model for the effective development of new small molecules for targeted therapy. Wei et al. well describe in their review the scientific effort in the development of TKi able to overcome resistance to crizotinib in ALK positive tumors [10]. Preclinical studies aimed at identifying the driver mutations in the target, but also in proteins belonging to associated pathways, is a fundamental step in developing new generation TKis.

4-one ring to rule them all: The broad spectrum importance of TKs in carcinogenesis determines that the same signaling pathway could be over-activated in different tumors. This is why some anti-TK families have been approved for different cancers. In addition, the use of a drug already approved in a different cancer offers the opportunity to speed up the approval process. KIT, PGF, LCK, FLT4 are among the most frequent targets in cancers (Figure 2). However, as described by Musumeci et al. for ponatibib, transferring targets between different tumors is not an obvious process [11]. Indeed, the multitarget characteristics of ponatinib and its high activity in haematological tumors, would suggest a potential use also in solid tumors. However, current clinical data have mainly shown a poor therapeutic value of ponatinib in cancer other that leukemia evidencing a scarce predictivity of preclinical models, and high risk of severe adverse effects. 

The path toward the cure of cancer with TKis has just started, and its building is dependent on the achievement of two goals in the close future: (1) more solid preclinical models able to recapitulate cancer heterogeneity in genetic background and expression profiles and (2) the definition of predictive markers for the effective design of multitarget therapy.

## Figures and Tables

**Figure 1:**
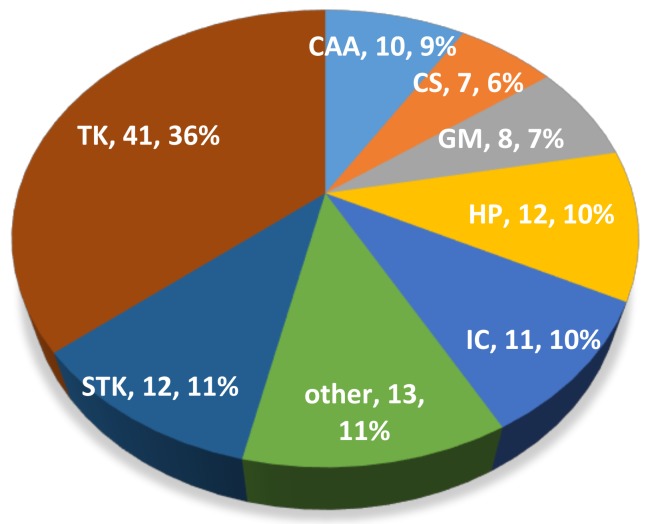
Targeted drugs approved for cancer were classified according to target type. TK: Tyrosine Kinase; STK: Serine Threonine Kinase; CAA: Cancer Associated Antigen; GM: Genome Maintenance; HP: Hormone Pathway; CS: Cytokine Signaling; IC: Immunological Checkpoint.

**Figure 2:**
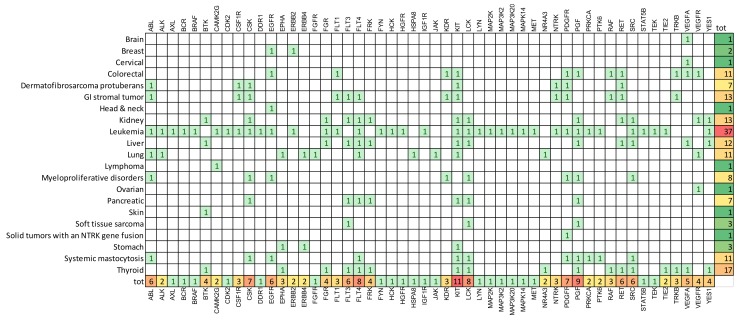
Tyrosine kinases targeted for different cancers by drugs approved in the clinic. The color gradient (from green to red) indicates the abundancy of the target for each cancer (right) or the frequency of the same target in different cancers (bottom).
